# SatEC: A 5G Satellite Edge Computing Framework Based on Microservice Architecture

**DOI:** 10.3390/s19040831

**Published:** 2019-02-18

**Authors:** Lei Yan, Suzhi Cao, Yongsheng Gong, Hao Han, Junyong Wei, Yi Zhao, Shuling Yang

**Affiliations:** 1 Technology and Engineering Center for Space Utilization, Chinese Academy of Sciences, Beijing 100094, China; yanlei@csu.ac.cn (L.Y.); gys@csu.ac.cn (Y.G.); hanhao17@csu.ac.cn (H.H.); weijunyong17@csu.ac.cn (J.W.); zhaoyi17@csu.ac.cn (Y.Z.); yangshuling17@csu.ac.cn (S.Y.); 2 Key Laboratory of Space Utilization, Chinese Academy of Sciences, Beijing 100094, China; 3 University of Chinese Academy of Sciences, Beijing 100049, China

**Keywords:** edge computing, on-board data processing, microservices, Integrated Terrestrial-Satellite Networks

## Abstract

As outlined in the 3Gpp Release 16, 5G satellite access is important for 5G network development in the future. A terrestrial-satellite network integrated with 5G has the characteristics of low delay, high bandwidth, and ubiquitous coverage. A few researchers have proposed integrated schemes for such a network; however, these schemes do not consider the possibility of achieving optimization of the delay characteristic by changing the computing mode of the 5G satellite network. We propose a 5G satellite edge computing framework (5GsatEC), which aims to reduce delay and expand network coverage. This framework consists of embedded hardware platforms and edge computing microservices in satellites. To increase the flexibility of the framework in complex scenarios, we unify the resource management of the central processing unit (CPU), graphics processing unit (GPU), and field-programmable gate array (FPGA); we divide the services into three types: system services, basic services, and user services. In order to verify the performance of the framework, we carried out a series of experiments. The results show that 5GsatEC has a broader coverage than the ground 5G network. The results also show that 5GsatEC has lower delay, a lower packet loss rate, and lower bandwidth consumption than the 5G satellite network.

## 1. Introduction

Fifth-generation (5G) Next Generation Communication Networks will be a global game changer from technological, economic, societal, and environmental perspectives [1]. In the future, 5G networks will be mainly used to meet the needs of enhanced mobile broadband services of approximately two billion users worldwide, as well as providing high reliability and low latency services in key communication scenarios [2]. The requirements of 5G networks for global coverage and low latency will have a great impact on the existing mobile network architecture of mobile operators.

The IMT-2020 (5G) Promotion Group defines “seamless wide-area coverage” as one of the four technical scenarios of 5G networks [3]. Terrestrial 5G communication requires ground base station support. Limited by economic costs, technology, and other factors, ground base stations cover only ~20% of the total land area. In desert, polar, and ocean regions, and other geographical locations where it is difficult to build the base stations, there are no communication networks. The wide-area coverage of a satellite radio access network could solve the coverage problem in 5G networks [4,5,6,7,8]. In recent years, advances in radar and laser communication technologies have made satellite communications more bandwidth, less cost, and lower latency [9]. O3b [10], OneWeb, SpaceX Starlink [11], and other low-orbit satellite constellations combine satellite communication services with Internet services, making it easier for users to connect to satellite networks.

However, in the development of traditional satellite communication, there is no unified framework and service interface, resource utilization is low, and computing power is poor. To provide services, satellites need to access ground computing centers to transmit data, which takes up a large amount of bandwidth and causes a high delay. In order to solve the delay problems, the ground 5G network uses Mobile Edge Computing (MEC) to place part of the computing resources at the edge of the network, which can effectively save bandwidth and reduce delay [12,13,14,15,16]. Considering the low latency requirements of 5G networks and the limited bandwidth resources of satellite networks, in this paper we propose a 5G Satellite edge computing framework (5GsatEC). In terrestrial 5G communication, edge computing already exists, but the concept of edge computing on satellites is rarely mentioned. In the past, the resources of computing, storage, and network in satellites were all developed for specific applications, rather than the universal edge computing that supports multiple applications.

There are three main contributions of our work. This paper presents the system framework of the 5GsatEC and its resource platform. The hardware of 5GsatEC is based on an embedded platform, and the software is decomposed into microservices. By using an embedded platform, 5GsatEC provides resources with low power consumption. Moreover, the platform has a wide variety of resources, and the optimal resource combination can be used to realize different services. Microservices will shield the underlying interface and provide available resources in the form of basic services [17,18]. Finally, the system characteristics of the ground network, satellite network, and 5GsatEC are simulated by MATLAB, which proves that the SatEC framework is highly feasible and can effectively guide the construction of practical applications.

This paper is organized as follows. Section 2 introduces the related research regarding satellite communication with 5G and satellite on-board computing. Section 3 introduces the SatEC system framework structure in terms of hardware and software. Section 4 reflects the system characteristics through a typical service for SatEC on the 5G satellite. Finally, Section 5 presents a conclusion based on the advantages and limitations of the SatEC system and offers a suggestion for future research.

## 2. Related Work

### 2.1. Satellite Communication with 5G

The satellite will be an important part of the next generation 5G communication network. Future satellite communication and terrestrial mobile communication systems will share the use of frequency resources and build a unified resource coordination platform [19,20]. In terms of hardware devices, 5G uses the development experience and results of Phased Array Antenna and Space Division Multiplexing (SDM) technologies in the field of satellite communications and the satellite industry to take advantage of 5G standardization, scale, and chip to reduce the high-frequency phased array antenna and improve reliability and integration. In addition, in terms of network control, the satellite network can be merged with the 5G virtualized core network through software gradual upgrade, and finally integrated into the 5G network control cloud in the form of satellite communication dedicated network element software and plug-ins [21].

The 5G context offers a promising opportunity to offer an integrated satellite/cellular service to 5G user equipment [1]. Earlier papers combined 5G and satellite communication through the Software Defined Network (SDN) technology [22,23,24]. Al-Zaidi and R et al. applied 5G base station nodes equipped with MEC capabilities to a marine data acquisition and cartography system over Ship Ad-hoc Networks (SANET) [25]. Z. Zhang et al. presented satellite network architecture based on mobile edge computing, which integrates network resources through a dynamic network virtualization technology and further designs a collaborative computing offload (CCO) model [26]. However, this work is more focused on the performance of task scheduling models, while our work specifies the design of the architecture.

### 2.2. Satellite On-Board Computing

With increasing satellite communication performance requirements, the satellite on-board data will depend more and more on the capability of the edge computing platform. Reconfigurable on-board processing using Field-Programmable Gate Array (FPGA) has been used in a flexible satellite communication system [27]. Additionally, some researchers have begun to study the application of advanced design concepts being used in on-board systems. OBC-NG is a reconfigurable on-board computing architecture for spacecraft [28]. It is based on a distributed heterogeneous network which incorporates Commercial Off-The-Shelf (COTS)-based processors and FPGAs. However, the above-mentioned system only considers the on-board application of devices or platforms. There is no consideration of how create the hardware and software resources of 5G satellite nodes in order to provide services to users. Therefore, it is necessary to design a framework for 5G Satellite edge computing and provide various kinds of specific services based on on-board computing resources and storage resources.

### 2.3. Drone Communications with 5G

In addition to satellites, drones are also considered as an important complement to ground 5G networks. Due to their flexibility and inherent ability for line-of-sight (LoS) communications, drone base stations (drone-BSs) can provide broadband, wide-scale, and reliable wireless connectivity during disasters and temporary events. Drone-BSs offer a promising solution for ultraflexible deployment and cost-effective wireless services [29]. Lagum et al. proposed a framework for strategic placement of multiple drone-BSs that provides wireless connectivity for a large-scale ground network [30]. M. Mozaffari et al. presented a delay-optimal cell association scheme in an unmanned aerial vehicle (UAV)-assisted terrestrial wireless network [31]. Mozaffari et al. proposed a concept of three-dimensional (3D) cellular networks and a framework for network planning for drone-BSs as well as latency-minimal cell association for drone user equipment [29]. Bor-Yaliniz et al. investigated both the mobile-enabled drones (MED) and wireless infrastructure drones (WID) cases within the realistic constraints of 5G and discussed potential solutions for highlighted open issues, either via application of current standards, or by providing suggestions towards further enhancements [32].

## 3. System Framework

### 3.1. System Design

The design of the SatEC framework needs to consider the characteristics of the satellite system, such as power consumption, reliability, scalability, and security. A specially designed hardware resource platform based on embedded computing platforms will be the best choice. Energy source limitation and heat dissipation of space-based systems limit the power consumption of on-orbit devices. Paolucci, P.S. et al. compared the power of nonembedded platform with that of embedded platform, the power consumption ratio is 14.375 [33]. Based on this result, we can conclude that the power consumption of SatEC is much lower than that of terrestrial edge computing platform. At the same time, compared with terrestrial 5G, services on satellite nodes are very different. It is necessary to design a new service system for 5GsatEC. The core idea of the system is the embedded high reliability hardware platform and the software architecture based on microservices. Furthermore, lightweight virtual mechanisms will be key to minimize the resources that the infrastructure requires.

**Figure 1:**
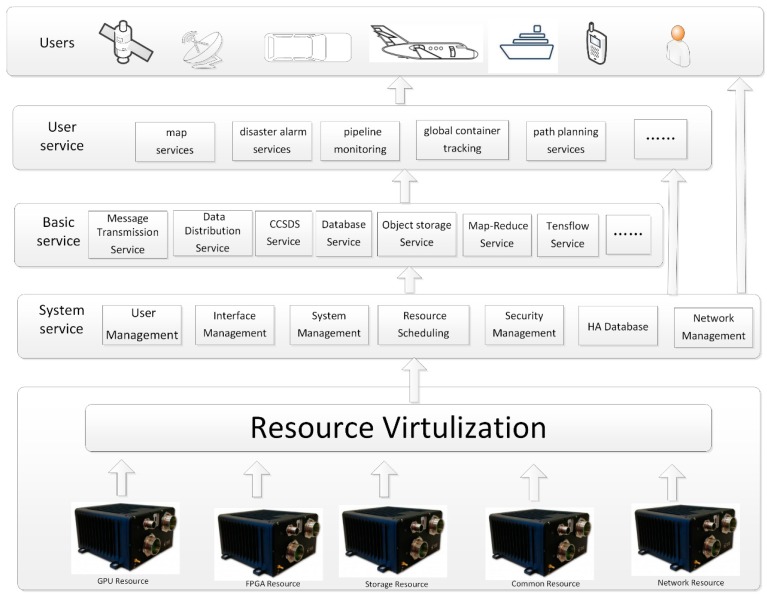
The framework of 5GsatEC.

The proposed design described in Figure 1 can be divided into two layers: the hardware platform and the software framework. The hardware platform, which is the infrastructure of the system, provides computing resource, storage resource, and network resource for services and users. The software framework is based on microservice architecture [18,34,35,36,37]. Using resource virtualization technology (virtual machine (VM), container, etc.), this layer shields the bottom hardware information and provides a uniform interface for users [38,39]. All the functions will be provided in the form of microservices. User’s services will be comprised by basic services.

### 3.2. Hardware Platform

**Figure 2:**
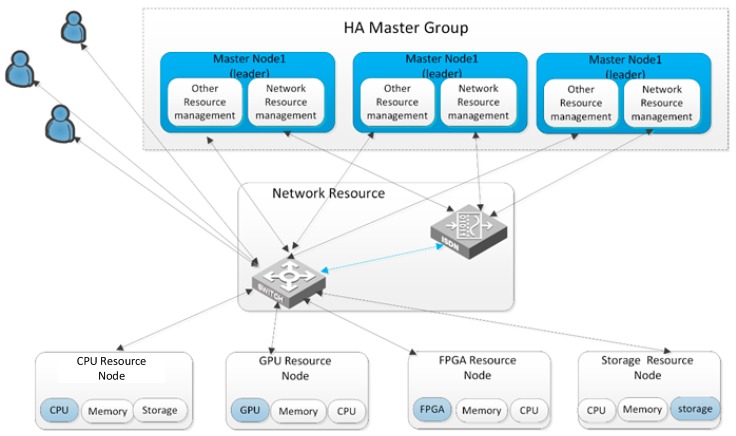
The framework of the resource platform.

All nodes in the hardware platform are built in an embedded platform. A typical choice is Xilinx Zynq UltraScale+™ MPSoC. The nodes in the hardware platform can be divided into two types: management nodes and resource nodes. Figure 2 shows the framework of the hardware resource platform. The management node is responsible for the user access, resource management and scheduling, service deployment and management, network scheduling, and security strategy, among other functions. It is the core of the whole system. Under management node control, the resource nodes support multiple types of device access. Basic resource nodes include central processing unit (CPU) nodes, graphics processing unit (GPU) nodes, FPGA nodes, storage nodes, and network resource. Meanwhile, the resource management interface is open to the user, and users can create resource nodes of new types easily. This framework not only improves the scalability of systems significantly, but also provides a friendly interaction for users.

1. Master node: The master node is the core of the system, so it is designed with high reliability systems. Multiple control nodes are deployed in the system, and the data consistency is guaranteed by a high availability database based on the paxos protocol [40]. There is only one leader node at a time. When the leader node is unavailable for some reason, the system automatically selects a new leader from the following nodes, allowing the system to keep running.
2. Common resource node: The common resource source nodes provide common resources such as CPU, memory, and storage to the SetEC system. Considering the power consumption constraints of on-board devices, the hardware system of these nodes is made of ARM CPU, which is based on the Reduced Instruction Set Computer (RISC) architecture. Compared with the Intel X86 processor, the ARM process has small instruction sets: the hardware logic is relatively simple. Therefore, it has fewer transistors than the X86 processor. The power consumption is consequently relatively low. Using container-based virtual technologies (such as Docker), common resource nodes can easily be used for virtualization.
3. GPU resource node: This kind of nodes provide GPU computing resources, which are mainly used in image processing, deep learning computation, and other compute-intensive applications. GPU resource nodes are based on GPU NVIDIA jetson chips. This series of chips contains several GPU cores and several ARM CPU cores. Using container virtual machines installed in the ARM CPU, the master node can control and schedule GPU resources.
4. FPGA resource nodes: This node provides FPGA computing resources for the system [41]. It is used for hardware acceleration in the fields of video/image processing, deep learning, gene detection, financial data analysis, and so on. The FPGA resource node is built on the Xilinx Zynq UltraScale + MPSoC FPGA platform, which contains multiple CPU cores and FPGA programmable logic (PL) resources. The PL logic can be dynamically reconfigured. Using container virtual machines installed in the ARM CPU, the master node can control and schedule FPGA programmable logical resources.
5. Storage resource nodes: The storage resources of SatEC are distributed in each node, and hard disks in all resource nodes can be used as storage resources in the SatEC resource platform.
  However, other types of resource nodes have limited storage capability. Their storage resource is mainly for their own use. The storage resource node is composed of ARM CPU and a large capacity solid-state hard disk, which is mainly used for the storage of massive data. Storage resource nodes support a variety of storage protocols: they can provide data access interfaces for files, objects, and data blocks. Database system can also be deployed in the storage resource nodes, and the database access interface is provided to the users.
6. Network resource nodes: The software defined network (SDN) is used in the management of the network resources, and the master node uses the OpenFlow protocol to manage network resource nodes. Based on the status of the network, a flow table is generated in the master node, and is sent to network resource nodes. The packets passing through the nodes will be routed according to rules in the flow table.
  Master nodes and other resource nodes are all connected to the network resource node. Data exchange between master nodes and resource nodes occurs through network resource nodes. Except for network resource nodes, a Representational State Transfer (REST) interface is used in resource management.

### 3.3. Software Framework

**Figure 3:**
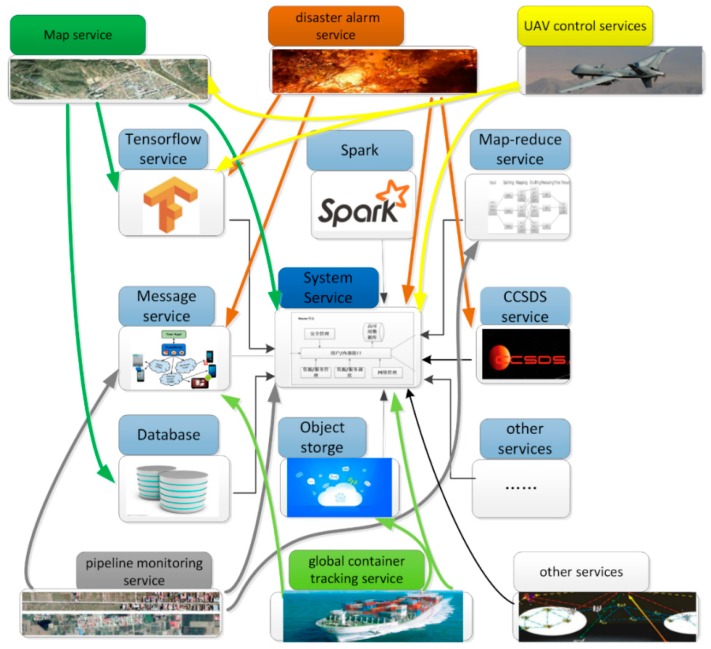
5GsatEC services.

We built a 5G satellite edge computing service architecture based on microservices for SatEC. According to the concept of “Software as a service” (SaaS), we built all the applications of the system on “service” [42]. SatEC services can be divided into three categories: system services, basic services, and user services. A system service is used for system management. It can provide services such as external/internal interface, resource/service management, resource/service scheduling, high availability database, and security management, and is the foundation of all other services. Basic services include some general functions that are open to external users. A user service is based on core services and basic services. It summarizes the common services of 5G satellite nodes at a higher level. Figure 3 shows the service architecture and typical services of 5GsatEC. End-users can use all services by way of the IP address: port number.

(1) System services: System services are consists of many components. These components are deployed in management nodes and different resource nodes. Components that will be deployed on the master node include: user management (including user registration and management services, user privilege management, etc.), interface management (API server, including the inside and outside interface), system management (resource node management/service management/service-resource mapping table/system log, etc.), resource scheduling, security management, network management, a high availability database, and so forth. The system service components that will be deployed in resource nodes are interface management (resource node API), container management components, and so on.

(2) Basic services: Typical basic services include message transmission services, data distribution services, Consultative Committee for Space Data Systems (CCSDS) services, database services, object storage services, Map-Reduce services, and TensorFlow services, among others. Basic services of the 5GsatEC are deployed by the system service through service images. Service images are stored in the image repository. Basic services are maintained by the system manager, and ordinary users only have the authority to use these services. Basic services can be extended, and also can be added or deleted when the system requirements change or the technology is upgraded. Basic services are always running.

Message transport service: Provides distributed messaging services based on a publish/subscribe mechanism. Messages can be divided into different topics. The producer releases messages, and the consumer reads messages by subscribing to topics.

Database services: Providing database services for massive data storage.

CCSDS services: Providing CCSDS protocol conversion, parsing CCSDS packets, and package data in the CCSDS protocol format.

Map-Reduce services: Services for big data computing, and also for networks resource control.

Spark services: A computing framework for data analysis.

TensorFlow services: Providing deep neural network services for video, image processing, and so on.

(3) User services: Typical user services include map services, disaster alarm services, unmanned aerial vehicle (UAV) control services, pipeline monitoring services, and global container tracking services. Most of the user services are developed by third party developers for specific applications of end-users. Service images are saved in the image repertory. When the user needs to use a service, it is deployed immediately with the service image, and when the user stops using the service, it will be deleted from the system.

Map services: Provide map services in remote areas that cannot be covered by 5G terrestrial base stations, such as mountains, oceans, and other areas. User information can also be collected at the same time.

Disaster warning services: release disaster alarm information to users in specific areas when disaster events are detected. Disaster messages can be sent by ground disaster warning agencies, and also can be identified automatically.

In 5GsatEC, the IP addresses of services change dynamically. In the process of system service expansion, failure recovery, and online upgrading, the running instances number of services is also dynamic. The user needs to obtain the available services and their invocation style through the service discovery mechanism. SatEC uses a highly reliable database to establish a service registry center at the master node to maintain the services list. The service registry provides service registration and query services through the REST API. POST\DELETE\GET are operations of REST API. The newly deployed services register their IP addresses and ports to the service registry through the POST operation. Services can be cancelled through a DELETE request. Through the GET operation, users can obtain information relating to available services.

## 4. Analysis, Comparisons, and Future Plans

### 4.1. Simulation Environment

In order to validate the proposed 5GsatEC framework, a test environment was built on the ground.

The full architecture, including hardware, software and services, will be verified in a prototype system in the future. The test platform will include eight nodes (Table 1). All these nodes will be developed in the embedded platform.

The key of the hardware platform in the 5GsatEC framework is the combination of heterogeneous embedded resources. Through the above demonstrated system, software based on microservices can shield the underlying hardware information, which can promote deployment of the framework in the 5G network.

**Figure 4:**
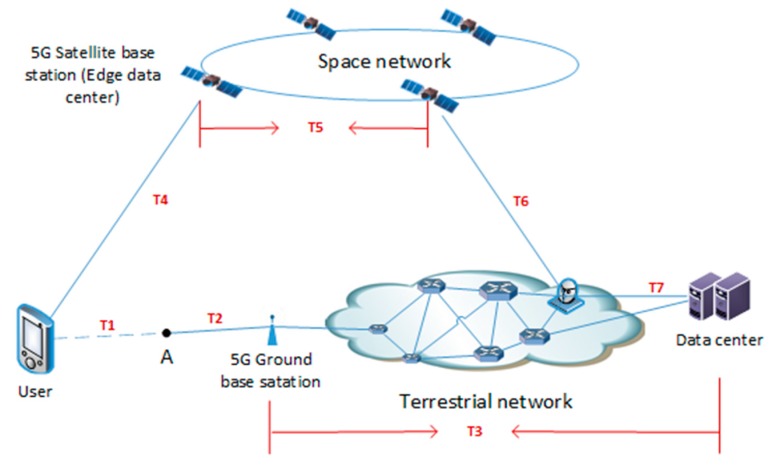
Experimental simulation scenario.

The advantages of the 5G satellite network over the ground 5G satellite network and existing satellite network are illustrated by two specific examples. The experimental simulation scenario is shown in Figure 4. Users in remote areas are not covered by the ground 5G communication network. Space networks are divided into the 5GsatEC network and satellite network, based on edge computing capabilities. In the simulation experiment, the satellite’s characterization parameters include the number of satellites, satellite height, processing power (calculation and storage), and number of supported users. The characterization parameters of the 5G communication network include coverage, number of nodes available, and number of supported users. The meaning of each time cost is explained in Table 2.

### 4.2. Simulation Results

Example 1: The user is located in an extremely remote area and wants to communicate with another user. Using the 5G ground network, users need to walk to point A at least because of the physical requirements for connecting to the ground base station, so the time delay of users’ access to the network is walking time T1+propagation time T2. However, if the 5GsatEC network is used, the total time delay is only T4, without needing to consider user movement. Although T4 may be larger than T2, it remains basically the at the same time scale as T2, and much less than T1.

According to the following settings, the time delays of the 5G satellite network and 5GsatEC network are compared. 5G satellite altitude: 500 km–1200 km [21]; ground 5G base station coverage: 300 m [43]; electromagnetic wave velocity: 3.0 × 108 m/s; distance from user to point A: 100 m; walking speed: 2 m/s. The delay between the ground 5G network and 5GsatEC is shown in Table 3. Although we compare the time delay, it actually shows the difference in the coverage performance of the ground 5G network and 5GsatEC networks. This is because timeliness is one of the characteristics of communication. When the delay exceeds a certain range, it is considered that the network cannot cover the area. Otherwise, when the delay was acceptable, the network was considered to have covered the area. In this experiment, the user can connect to the ground base station by his own movement, but the walking time is much longer than the ordinary communication time. It has bad timeliness. In fact, the ground network has no coverage for this user. We illustrate network coverage performance through the comparison of delay. It is a conversion from qualitative problem to quantitative problem.

**Figure 5:**
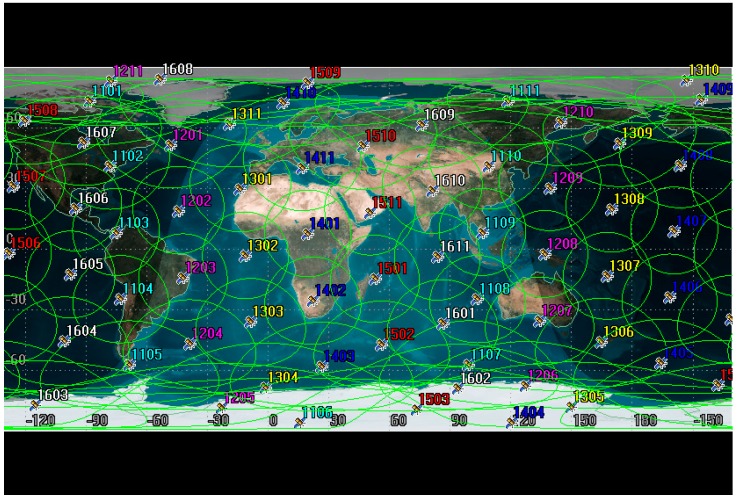
Geographic coverage of Iridium satellite.

**Figure 6:**
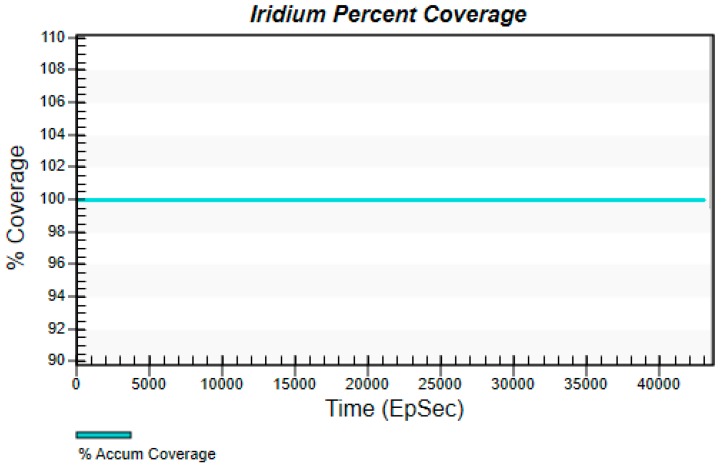
Iridium percent coverage.

**Figure 7:**
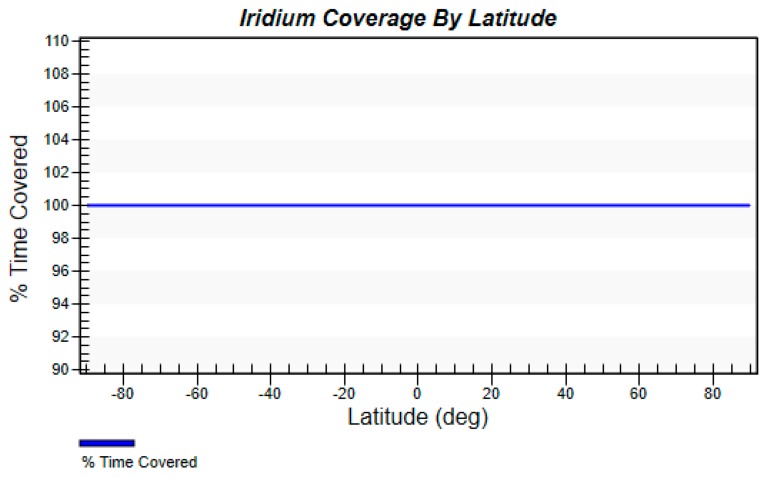
Iridium coverage by latitude.

In addition, we refer to the constellation model of the Iridium communication system [44] and test the satellite communication coverage through the (Satellite Tool Kit) STK simulation software, as shown in Figure 5, Figure 6 and Figure 7. Figure 5 shows a plan view of the global coverage of satellite networks.

Figure 6 and Figure 7 are obtained by calculation of STK’s coverage analysis tool. Figure 6 shows that at each moment, the accumulative coverage of the satellite to the global region is 100%. Figure 7 shows that at different latitudes, the satellite coverage time ratio is 100%. Through the simulation of satellite coverage performance, it can be found that setting a reasonable satellite constellation can make up for the shortcomings that the network is difficult to cover in remote areas.

Example 2: Users are located in extremely remote areas and want to achieve cloud processing of application data (suppose there is a lot of data to be processed, but the amount of data returned is very small, so the transmission delay of the return can be neglected). If traditional satellite communication is used, the data need to be sent to the remote data center. The total delay for the user to obtain the result is T4+T5+T6+T7+data processing delay. However, if the 5GsatEC network is used, the user data can be processed in the edge data center. The total delay to obtain the user’s results is T4+data processing delay, which is obviously better than the network framework without edge computing [5,45,46].

According to the following settings, the delay of the 5G ground network and 5GsatEC network is compared. 5G satellite base station upstream rate: 10 Mbps; downstream rate: 100 Mbps [3]; user data quantity: 400 M; ground data processing time: 4 s; on-board data processing time: 7 s; transmission delay of intersatellite link: 2 s; transmission delay of ground network: 0.1 s. The packet loss rate per hop in the network transmission is 1/1000, and the packets will be retransmitted until they reach the destination.

**Figure 8:**
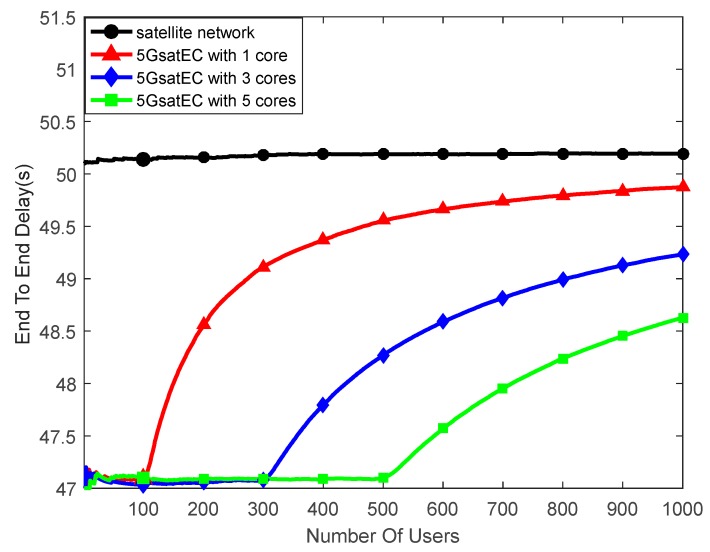
Delay between the satellite network and 5GsatEC with no storage.

According to the above parameters, we built a model in MATLAB. In this paper, we focus on analysis of the computation capacity and the storage capacity of the proposed framework. The delay varies with the number of users, as depicted in Figure 8. We assume each process mode has no storage resource in this case. Each user occupies a part of the computation resource. When the on-board computing resources are fully occupied, the data will be unloaded to the ground computing centers, which is the same as in the traditional satellite communication mode.

The results show that when the framework computes with one core, it can provide a low latency service within 100 users. Even if there are more than 100 users, the average delay is still smaller than when using the satellite network. 5GsatEC optimizes the calculation delay in the edge area, and the number of users reducing latency is positively correlated with the on-board computing capacity.

**Figure 9:**
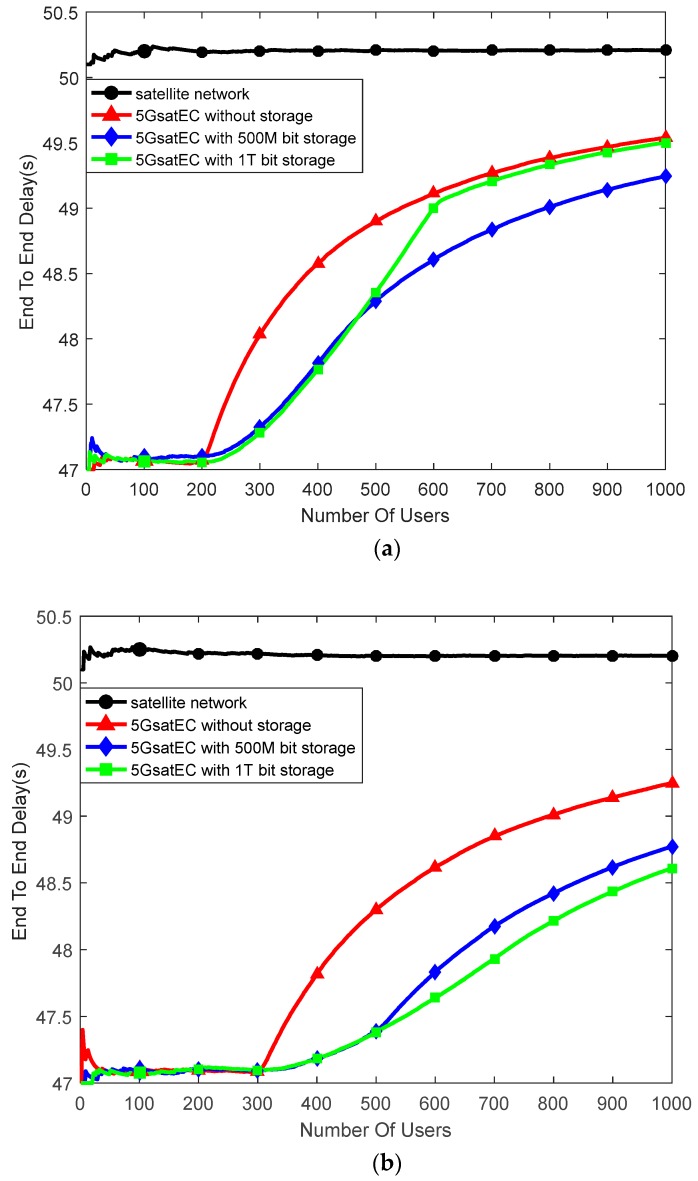
Delay between the satellite network and 5GsatEC. (**a**) Two computing cores with different storage capacities and (**b**) three computing cores with different storage capacities.

Figure 9 shows the relationship between end-to-end latency and storage. In the 5GsatEC framework, when computing resources are fully occupied, the computing tasks will be stored at first and then computed later. Task queuing duration is related to computing capacity. 

In Figure 9a, we set the 5GsatEC computing resource as two cores. When storing 500 M bit tasks, the total queuing and processing time is longer than the processing time through the satellite network, and the performance of storing 1 T bit tasks is lower than that of storing 500 M bit tasks. However, the average end-to-end delay of 5GsatEC is still lower than that of the satellite network. 

In Figure 9b, the 5GsatEC computing resource is set to three cores, and the stronger computing capacity reduces the queuing delay, so that the delay decreases with the increase of storage capacity. Figure 9 shows that the optimal value of the cache task is related to the computing capacity. When the average queue time lower than the time difference between the processing time of the satellite network and that of 5GsatEC, the delay decreases as the storage capacity increases.

**Figure 10:**
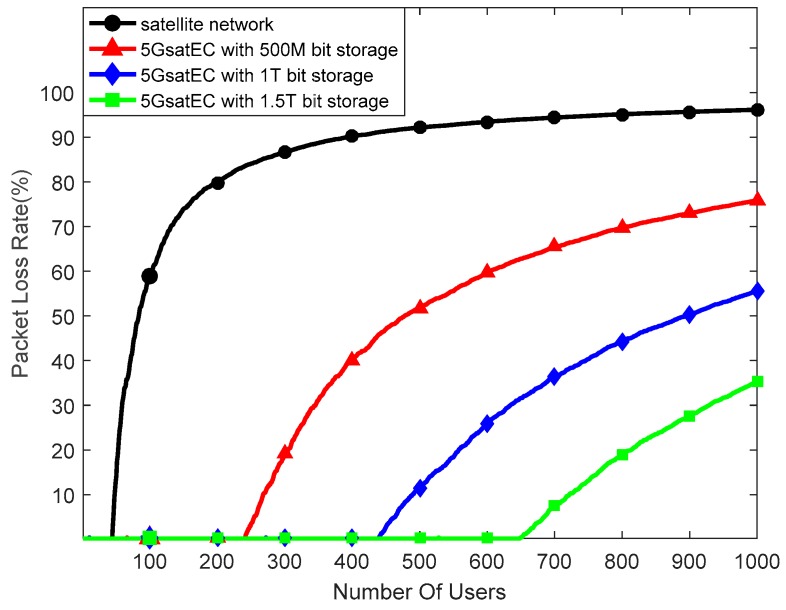
Packet loss rate between the satellite network and 5GsatEC.

Figure 10 shows the relationship between the packet loss rate and storage resource in the condition of increasing users. In this case, we set the 5GsatEC computing resource as one core. In the satellite network, when the number of users exceeds the system forwarding capability, the satellite network will discard excess packets, causing the packet loss rate to increase rapidly. In the 5GsatEC framework, when the rate of receiving data packets is greater than its processing capacity, 5GsatEC will store the redundant data packets and process them later to reduce the packet loss rate. 

As shown in Figure 10, the packet loss rate decreases with the increase of storage capacity in the 5GsatEC framework, and the performance with a decreasing packet loss rate is significantly better than the performance achieved by the satellite network.

**Figure 11:**
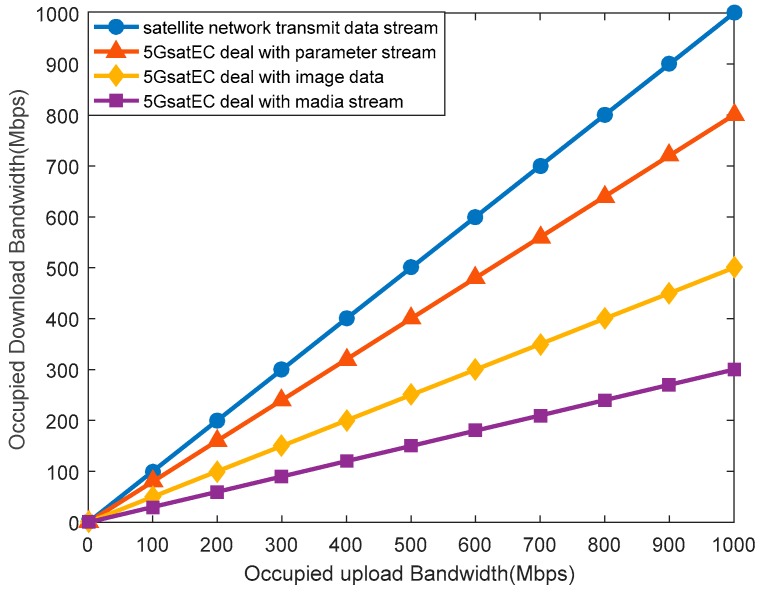
Occupancy differences between the satellite network and 5GsatEC.

Figure 11 depicts the relationship between upload bandwidth occupancy and download bandwidth occupancy. The satellite network simply forwards data packets, so the upload bandwidth occupancy is the same as the download bandwidth occupancy. However, the packets will be processed before they are forwarded in 5GsatEC. Different data types have different degrees of compressibility. 

Figure 11 shows the downstream bandwidth occupancy of the satellite network and 5GsatEC when they deal with different data packets. The 5GsatEC saves a significant amount of download bandwidth compared to the satellite network.

5GsatEC has the characteristic of broader coverage, like the satellite network; meanwhile, on-board data processing and storage make up the long end-to-end delay. Service performance in 5GsatEC is related to storage and computing resources. More storage and computing resources can be placed on board as weight and power consumption permit.

## 5. Conclusions

In this article, we have presented a novel framework—SatEC—for providing services using 5G satellites. In this framework, all resource nodes are embedded, and they are virtualized into a resource platform for deploying services. All functions of this framework are decomposed into services. There are three kinds of services: system services, basic services, and user services, and they play different roles in the framework. Basic services and user services can be deployed to resource platforms by the system service. Compared to other frameworks, the SatEC framework has the characteristics of broader coverage, lower user latency, a lower packet loss rate, and lower bandwidth consumption.

In the future, we will build a prototype system with eight embedded resource nodes. Lightweight platform software and a specially designed resource allocation algorithm will be developed to improve service performance. We will also explore the possibility of using more kinds of high-performance embedded platforms in the resource nodes. Finally, more services for 5G satellite users will be deployed on this platform.

## Figures and Tables

**Table 1:** Resources in the test platform.

Nodes	Quantity	Remarks
Master node	3	Also used as common resource nodes
Common resource node	1	-----
FPGA resource nodes	1	-----
GPU resource node	1	-----
Storage resource node	1	-----
Network resource nodes	1	-----

**Table 2:** The meaning of each time cost.

Time Cost	Meaning
T1	The time cost of moving to the coverage of communication networks
T2	The time cost of forwarding data in terrestrial 5G network
T3	The time cost of processing data in terrestrial 5G network
T4	The time cost of uploading data
T5	The time cost of forwarding data among intersatellite links
T6	The time cost of downloading result
T7	The time cost of forwarding data to data center

**Table 3:** Delay between ground 5G network and 5GsatEC.

Network Type	Ground 5G Network	5GsatEC
Delay (s)	50.0000	0.0016
